# High-Sensitive Troponin Measurement in Emergency Department Patients Presenting with Syncope: A Retrospective Analysis

**DOI:** 10.1371/journal.pone.0066470

**Published:** 2013-06-18

**Authors:** Gregor Lindner, Carmen A. Pfortmueller, Georg-Christian Funk, Alexander B. Leichtle, Georg Martin Fiedler, Aristomenis K. Exadaktylos

**Affiliations:** 1 Department of Emergency Medicine, Inselspital, University Hospital Bern, Bern, Switzerland; 2 Department of Internal Medicine, Inselspital, University Hospital Bern, Bern, Switzerland; 3 Department of Respiratory and Critical Care Medicine, Otto Wagner Spital Vienna and Ludwig Boltzmann Institute for COPD and Respiratory Epidemiology, Vienna, Austria; 4 Center for Laboratory Medicine, Inselspital, University Hospital Bern, Bern, Switzerland; King’s College London School of Medicine, United Kingdom

## Abstract

**Objective:**

To study the relevance of high-sensitive troponin measurements in the acute workup in patients admitted to the emergency department of a large university hospital due to syncope.

**Methods:**

In this retrospective study all patients admitted to the emergency department because of syncope of the Inselspital, University Hospital Bern between 01 August 2010 and 31 October 2012, with serial determination of high-sensitive troponin (baseline and three hours control) were included. Of all identified patients we obtained data on demographics, laboratory data, ECG as well as on outcome. A change in high-sensitive troponin in the three hours control of +/−30% compared to baseline was considered significant.

**Results:**

A total of 121 patients with a mean age of 67 years (SD 16) were included in the study. 79 patients (65%) were male and 42 (35%) were female. There was no significant difference in the median high sensitive-troponin level at baseline and in the three hours control (0.01 mcg/L [0.003 to 0.022] versus 0.011 mcg/L [0.003 to 0.022], p = 0.47). Median percent change in high-sensitive troponin level between baseline and control was 0% (−9.1 to 5). 51 patients (42%) had elevated high-sensitive troponin levels at baseline with 7 patients (6%) showing a dynamic of +/−30% change from the baseline measurement in the 3 hours control. 3 of these patients received coronary angiography due to the dynamic in high-sensitive troponin, none of whom needed intervention for coronary revascularization.

**Conclusions:**

On basis of the current study, where no single patient took benefit from determination of high-sensitive troponin, measurement of cardiac troponins should be reserved for patients with syncope presenting with symptoms suggestive for the presence of an acute cardiac syndrome.

## Introduction

In the Guidelines of the European Society of Cardiology, syncope is defined as a transient loss of consciousness caused by a self-limiting global cerebral hypoperfusion characterized by rapid onset, short duration, and spontaneous complete recovery [1]. About 1 to 3% of admissions to the emergency department (ED) and 6% of hospital admissions are due to syncope [2]. While many cases of syncope have a benign cause, syncope under certain circumstances is an alarming sign for serious underlying pathologies such as severe aortic stenosis or cardiac arrhythmias [3]. Cardiac syncope is associated with the worst prognosis with a 1-year mortality of 10–30% while vasovagal or situation syncope is not associated with an increased mortality [4], [5]. Current guidelines on the evaluation for syncope exist from the large cardiologic societies and are freely available online. Although neither the guidelines of the American Heart Association nor those of the European Society of Cardiology recommend tests for high-sensitive troponin in patients presenting with syncope, cardiac troponins are often determined in these patients in daily clinical practice, despite syncope is not regarded a classic symptom of acute coronary syndrome. Thus, we hypothesized that serial determination of high-sensitive troponin to detect unstable coronary artery disease as an underlying cause of syncope does not result in a benefit in the workup of patients presenting to an ED due to syncope.

So far only one clinical study has determined the role of high-sensitive troponin in the evaluation of syncope [6]. Surprisingly, the authors found that the majority (77%) of the 338 included patients had troponin levels above the limit of detection. 36% of patients had a high-sensitive troponin level above the 99^th^ percentile of the reference population. However, the study did not answer the question in which way a determination of troponin is of any benefit for the patient in the workup of syncope.

In the present study we aimed to determine the relevance of serial high-sensitive troponin determination in patients presenting to a large emergency department of a university hospital. Additionally, we wanted to identify the number of patients with acute myocardial infarction detected by serial high-sensitive troponin measurements in patients admitted to an ED for syncope.

## Methods

### Ethics Statement

The study was approved by our local institutional review board (Ethics Comission of the Canton of Bern, Switzerland – www.kek-bern.ch). The need for written consent was waived by our institutional review board due to the retrospective design of the study.

In this retrospective study, we screened the database of the Department of Emergency Medicine of the Inselspital, University Hospital Bern, a large university hospital, in order to identify all patients presenting with syncope. Of all patients presenting with syncope, those with serial measurements of high-sensitive troponin as determined during the stay in our ED between 01 August 2010 and 31 October 2012 were included.

High-sensitive troponin was measured by the Center for Laboratory Medicine using the Roche Modular E170, an electrochemiluminescence immunoassay. The reference range of high-sensitive troponin in our laboratory is defined as <0.014 mcg/L, equivalent to the 99^th^ percentile of high-sensitive troponin in the normal population. The limit of detection of high-sensitive troponin in our laboratory equals 0.003 mcg/L.

Of all identified patients we gathered demographic data including age and sex as well as data on the admission cause, cardiovascular risk factors including known coronary arteries disease, diabetes, arterial hypertension, dyslipidemia, history of smoking, cardiovascular family history and adipositas. Additionally, we obtained information on the medication presently taken by the patients, ECG and laboratory data (high-sensitive troponin levels, electrolytes, c-reactive protein and creatinine levels. Data on outcome including hospitalization rate, hospital length of stay and in hospital mortality were registered in the database.

On basis of serial high-sensitive troponin measurements we calculated percent changes between the second and the first determination. A rise or fall exceeding or equaling 30% of the baseline high-sensitive troponin measurement was considered significant [7].

Results are presented as means and standard deviation (SD) or median and first and third quartile, as appropriate. Wilcoxon matched pairs test was used to compare serial high troponin measurements. A p-value <0.05 was considered to be significant.

## Results

During the study period a total of 55.422 patients were admitted to the ED. Of these, 1.218 were admitted for syncope. 121 patients had serial measurements of high-sensitive troponin and were included in the study. Mean age of patients was 67 years (SD 16). 79 patients (65%) were male and 42 (35%) were female. 4 patients (3%) presented with minor head trauma along with the syncope.

**Figure 1:**
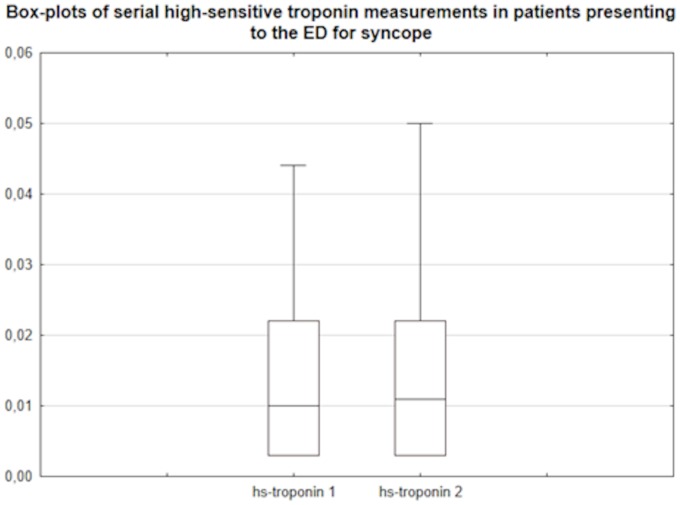
Box-plots of serial high-sensitive troponin measurements in mcg/L. While hs-troponin 1 is the baseline determination and hs-troponin 2 is the 3 hours control.

8 patients (6%) complained of chest pain on admission to the ED. 41 patients (34%) had abnormalities in the ECG: 19 patients (46%) showed ST segment depression, 8 (20%) right bundle branch block, 5 (12%) a negative T wave, 3 (7%) a left bundle branch block, 2 (5%) presence of Q-waves and bifascicular block, respectively. Tachycardic atrial fibrillation and atrioventricular block grade I was present in 1 patient (2%), respectively. All patients received a baseline measurement of high-sensitive troponin in the ED and a control measurement three hours after ED admission. Median high sensitive-troponin level at baseline was 0.01 mcg/L (0.003 to 0.022) and three hours control was 0.011 mcg/L (0.003 to 0.022). Table 1 gives an overview on the high-sensitive troponin distribution at baseline and the three hours dynamic. There was no difference between the baseline measurement and the three hours control (0.01 vs 0.011 mcg/L, p = 0.47). Median change in high-sensitive troponin level between baseline and 3 hours control measurement was 0 mcg/L (−0.001 to 0.001; Figure 1). Median percent change in high-sensitive troponin level between baseline and control was 0% (−9.1 to 5).

**Table 1:** Overview of high-sensitive troponin distribution and dynamic at the three hours control.

Baseline high-sensitive troponin	Number (%)	Rise to ≥0.014 mcg/L in 3 hours control, number (%)	Change in high-sensitive troponin at 3 hours control of ±30%
<0.003 mcg/L	37 (31%)	0 (0%)	5 (14%)
0.003–0.013 mcg/L	33 (27%)	4 (12%)	8 (24%)
≥0.014 mcg/L	51 (42%)	−	7 (14%)

70 patients (58%) had a high-sensitive troponin measurement of <0.014 mcg/L at baseline, while 4 of them had increases to a level equaling or exceeding the cutoff for the normal range of 0.014 mcg/L at the control measurement. However, only in one of these patients a significant troponin rise exceeding 30% of the baseline value was found (790% rise or from 0.01 to 0.89 mcg/L). The patient was found to suffer from intracranial bleeding, finally ending lethal.

51 patients (42%) had elevated high-sensitive troponin levels at baseline with 7 patients (6%; only one presenting with chest pain) showing a dynamic of +/−30% change from the baseline measurement in the 3 hours control. In 3 of the patients with a high-sensitive troponin dynamic exceeding 30% from baseline to control no none-cardiac cause could be attributed to the rise in troponin and they finally received coronary angiography. However, none of the patients was found to have a significant stenosis in the coronary angiography leaving the cause for the elevated troponin unclear. None of the 7 patients with a high-sensitive troponin change of +/−30% from baseline measurement died.

No single patient included in our study finally received the diagnosis of acute coronary syndrome. One patient (1%) died during hospitalization from intracerebral hemorrhage.

## Discussion

In the present study, we found that 121 patients received measurement of high-sensitive troponin during their initial evaluation at the ED for syncope. Although 42% of patients showed initial high-sensitive troponin levels of ≥0.014 mcg/L, only 6% had a significant high-sensitive troponin dynamic of +/−30% at the 3 hours control measurement. 3 of the patients received coronary angiography, all of which ended without need for intervention.

Although troponin measurements are not recommended in the current guidelines for the evaluation of syncope of the large societies, it is often performed by physicians in daily clinical practice [1], [3], [8]. Reed an coworkers studied high-sensitive troponin measurement in 338 patients admitted to an ED for syncope [6]. High-sensitive troponin was measured 12 hours after the syncope. The authors found that patients with elevated high-sensitive troponin results had a significantly increased probability of reaching the endpoint of a serious outcome including death [6]. Sun and colleagues in a similar study found that an elevated conventional troponin I was associated with adverse 30-day outcome in patients of 60 years or older after presentation for syncope to an ED [8]. However, both studies left open the question on how this information does alter management of patients presenting with syncope. The present study showed that determination of high-sensitive troponin levels in patients presenting with syncope to an ED did (as could be expected) not result in detection of unstable coronary artery disease as the cause of syncope. We do not have data on long term outcome of patients, but it should be noted that it was not our intention to (repeatedly) show that high-sensitive troponin elevation is associated with adverse outcome, but to analyze whether its determination is of any benefit for the patient in the acute workup for syncope.

It is well described today that various patient populations with an elevation of high-sensitive troponin are at risk for adverse outcome [9]–[11]. However, the use of high-sensitive troponin measurement in the evaluation of syncope is not clear so far. In our population no single patient was diagnosed with acute coronary syndrome in the ED. Three patients in our cohort received coronary angiography due to a dynamic in serial high-sensitive troponin measurements. However, none of the patients needed an intervention for revascularization. On basis of the current data a measurement of high-sensitive troponin cannot be recommended in the routine evaluation of patients admitted to the ED for syncope. Results of the high-sensitive troponin measurement might be elevated for several reasons: Hypertensive crisis, heart failure, pulmonary embolism, renal insufficiency, blunt chest trauma, aortic dissection or even strenuous exercise can lead to elevated high-sensitive troponin levels [12]. Receiving such elevated high-sensitive troponin levels will then bring the physician into a dilemma of how to proceed often ending in serial measurements without trend-setting results.

But there is also a financial aspect: Currently one determination of high-sensitive troponin in our institution costs the amount of 23 Swiss Francs (approximately 24 United States Dollar). The routine determination of high-sensitive troponin in a common (and often benign) condition such as syncope would result in serious amounts of unnecessary health care spending.

Our study is limited by its retrospective and single site design. The detailed thoughts behind ordering high-sensitive troponin determination of the physician in charge are unknown. Additionally, we do not have information on long-term outcome of patients. On the other hand, long term outcome is not necessary in order to answer our study question.

In conclusion, based on our study in which no single patient took benefit from the determination of high-sensitive troponin, routine measurements of cardiac troponins cannot be recommended for the evaluation of syncope in the ED. In the workup for syncope, the measurement should be reserved for patients who present with symptoms suggestive for the presence of acute coronary syndrome.
